# Compulsory hospitalization in child psychiatry: clinical and sociodemographic profile of Tunisian inpatients

**DOI:** 10.1192/j.eurpsy.2023.480

**Published:** 2023-07-19

**Authors:** L. Sahli, Z. Abbes, S. Halayem, A. Bouden

**Affiliations:** child and adolescent Psychiatry, Razi Hospital, Manouba, Tunisia

## Abstract

**Introduction:**

The department of child psychiatry in Razi Hospital is the unique psychiatric unit in Northern Tunisia offering full-time hospitalization for minors with mental health disorders.

**Objectives:**

The aim of our study was to explore the clinical and therapeutic characteristics in compulsory admissions of minors in the department of child psychiatry in Razi hospital between 2011 and 2019.

**Methods:**

We conducted a retrospective study of medical records of inpatients admitted to the hospitalization unit of the child psychiatry department in Razi Hospital in Tunisia between 2011 and 2019.

**Results:**

Over the nine years, the total number of compulsory admissions was 74 inpatients, aged from 11 to 16 years old. The number of compulsory admissions increased from 03 in 2011 to 22 in 2019. Most of the inpatients were boys (sex ration=1.46). The mean age was 14, 1 year old. Heteroagressiveness was the reason for admission in half of the cases followed by risk behaviors (30.1%) and suicidal behaviors (18.6%). The diagnosis of conduct disorder was found in 33.3% of the cases followed by a normal psychiatric examination in 11.8% of the cases. Mood disorders were found in 9.8% of the cases. Parental psychoeducation (100%), individual psychotherapy (91%) and family therapy (88,2%) were the treatment of choice for the inpatients. The prevalence of psychiatric medication was 45.1%. The most important forms of medication used were neuroleptics (42.3% of medicated patients) and mood stabilizers (30.7%). Child protection delegates were involved in 86,4% of the cases for social intervention.

**Conclusions:**

Minors admitted in an involuntary mode to psychiatric unit have their own specifities in terms of clinical and therapeutic characteristics. More theoretical and empirical research is needed regarding the involuntary admission of minors.

**Disclosure of Interest:**

None Declared

